# The Constructional Approach to Zoo Animal Training: Enhancing Welfare Through Emerging Evidence-Based Behavioral Science

**DOI:** 10.3390/ani15213221

**Published:** 2025-11-06

**Authors:** Barbara Heidenreich, Annette Pedersen

**Affiliations:** 1 Animal Training Fundamentals, Austin, TX 78745, USA; 2 Copenhagen Zoo, Roskildevej 38, 2000 F, Denmark; ap@zoo.dk

**Keywords:** animal training, animal welfare, constructional approach, functional assessment, positive reinforcement, negative reinforcement, genuine choice, nonlinear contingency analysis, assent, compassion

## Abstract

**Simple Summary:**

Animal welfare has become a cornerstone of modern zoo and aquarium animal care practices. This article introduces the constructional approach to animal training as a practical, emerging evidence-based method that can improve zoo animal welfare. Unlike traditional training approaches that focus on eliminating problem behaviors, the constructional approach emphasizes building desirable behaviors, providing animals with genuine choices, and understanding their emotional welfare through analyzing contingencies. This review explains the core principles of the constructional approach, highlights its advantages over hierarchical models of behavior change, and provides practical examples of successful implementation in zoo settings. By implementing constructional programs, zoos can achieve their welfare goals more effectively while fostering compassionate relationships between animals and caregivers. The constructional approach represents an advancement in animal training, providing a framework that integrates scientific rigor with ethical care practices to benefit both animals and the professional communities that care for them.

**Abstract:**

Animal welfare has become a cornerstone of modern zoo and aquarium animal care practices. This paper introduces the constructional approach to animal training as an evidence-based framework that can enhance the welfare of zoo animals. Developed through decades of behavioral science research and practical applications, the constructional approach emphasizes building desirable behaviors rather than eliminating problematic ones, avoiding reduction-based techniques, utilizing comprehensive contingency analysis, incorporating genuine choice, and addressing emotional welfare through contingency management. This review systematically examines the foundational principles of the constructional approach, distinguishes it from traditional animal training methodologies, presents case examples of successful implementation in zoo settings, and provides practical recommendations for zoo professionals. Methods included a narrative review of peer-reviewed literature, unpublished academic works, and documented applications in zoological settings. The results demonstrate that constructional programs offer notable advantages over commonly promoted hierarchical models of behavior change procedures, which often prescribe sequential application of techniques without adequate consideration of behavioral function. By adopting constructional programs, zoos can more effectively meet their overriding goals of providing optimal welfare, supporting conservation efforts, facilitating research, and enhancing educational experiences—all while prioritizing compassionate care that respects the agency and well-being of animals.

## 1. Key Concepts and Definitions

The following are foundational terms and concepts frequently referenced in discussions of the constructional approach. They are introduced here to support the reader’s understanding and will be examined in greater depth throughout this manuscript.

**Appetitive:** An appetitive is a stimulus, condition, or event that an animal will approach, contact, or maintain proximity to. When delivered under specific stimulus conditions following a behavior, it can increase the likelihood of that behavior occurring in the future. Whether an appetitive functions as a reinforcer depends on the stimulus conditions present and the animal’s learning history.

**Aversive:** An aversive is a stimulus, condition, or event that an animal will attempt to escape, avoid, or reduce contact with. When removed under specific stimulus conditions following a behavior, it can increase the likelihood of that behavior occurring in the future. Whether an aversive functions as a negative reinforcer depends on the stimulus conditions present and the animal’s learning history.

**Positive Reinforcement:** Positive reinforcement is a learning process in which a stimulus, event, or condition is added following a behavior, and this relation results in an increased likelihood of that behavior occurring again under similar conditions. It is also applied as a procedure in which stimuli are arranged to follow behavior, but the specific form this takes may vary depending on the setting, the animal, and the outcomes being arranged.

**Negative Reinforcement:** Negative reinforcement describes a learning process in which behavior increases because it results in the removal or avoidance of a stimulus, event, or condition. As a procedure, it involves arranging contingencies so that stimuli are removed or avoided contingent on behavior, with specific applications differing according to context and the desired outcomes.

**Contingency:** A contingency describes the conditions under which behaviors occur and the outcomes they may produce. This is a simplified explanation to introduce the concept; contingencies will be explored in greater depth in later sections, particularly within the framework of Nonlinear Contingency Analysis (4.3.2).

For example, when a stimulus, such as a syringe, is presented to an animal (conditions), if the animal backs away (behavior) and gains distance from the syringe (outcome), this describes a contingency that may maintain behavior through negative reinforcement. By analyzing and modifying such contingencies rather than merely treating symptoms that may be labeled as fear, the constructional approach fosters lasting behavioral change that enhances welfare.

**Distancing and Nearing Contingencies:** Distancing and nearing contingencies are two fundamental types of contingencies that maintain behavior. Distancing contingencies involve behaviors that increase distance from aversive stimuli (maintained by negative reinforcement), while nearing contingencies involve behaviors that decrease distance from appetitive stimuli (maintained by positive reinforcement). Understanding whether an animal’s behavior is maintained by distancing or nearing helps determine appropriate intervention approaches.

**Critical Consequence:** A critical consequence is an outcome that maintains behavior because it is especially valued by the individual under the current conditions. It is the consequence that governs when and why certain behaviors are emitted. In constructional terms, the focus is not just on identifying what that consequence is but also recognizing that there may be multiple behaviors or patterns of behavior that produce access to it. Rather than assuming a single behavior leads to a single result, constructional thinking involves analyzing the broader set of behaviors that can lead to that important outcome. This perspective allows trainers to shape new, functional behaviors that help animals achieve what matters to them most.

**Degrees of Freedom:** The number of ways an animal can obtain a critical consequence, minus one. When animals have multiple pathways to access important reinforcers (both positive and negative), they experience less coercion and greater agency. This is described further in later sections. 

**Superimposition:** When one contingency is layered on top of another. This concept, explored through Nonlinear Contingency Analysis (4.3.2), explains why some traditional intervention approaches may appear successful temporarily but fail to create lasting change.

**Emotional Behavior:** Observable behaviors such as approach/avoidance responses, body postures, vocalizations, and physiological changes that can be measured and used to describe emotional states. Rather than relying on subjective emotional labels (e.g., “fear” or “confidence”), careful observation and analysis of these behaviors and their environmental context can inform our understanding of the contingencies in place.

**Occasion:** Occasioning conditions refer to the specific environmental or contextual variables present just prior to a behavior’s emission, which consistently signal the availability of reinforcement. Not all antecedents occasion behavior, but those that reliably do are considered occasioning stimuli or stimulus conditions.

**Potentiate**: To potentiate a behavior or consequence means to alter observable environmental conditions to increase the likelihood that a particular behavior will occur or that a consequence will function effectively as a reinforcer. The term is preferred within the constructional approach because it reflects measurable, functional relationships between behavior and the environment. In contrast, the word “motivate” suggests internal states or drives that cannot be directly observed or manipulated, making it less precise and not actionable for behavior analysis. Potentiation allows practitioners to focus on arranging conditions that support behavior change without relying on inferred explanations.

These initial definitions are intended to provide a framework for the reader, setting the stage for more detailed discussions of how these concepts are applied within the constructional approach to support sustainable behavior change and improved welfare in animal training.

## 2. Introduction

Ensuring the highest possible animal welfare standards has become a key priority for modern zoological institutions [1,2]. As noted by Talló-Parra and Manteca [3] in their call for papers, “to thrive, zoos should embrace animal welfare as a guiding principle, the pathway through which they achieve their institutional goals, and a fundamental end in itself” (p. 1). This paradigm shift requires evidence-based approaches that can be systematically applied across diverse taxa and contexts within zoological settings [1].

The constructional approach to behavior change, first described by Israel Goldiamond in 1974 [4] represents a comprehensive and compassionate framework that aligns with contemporary understanding of animal welfare science. Although developed initially within human clinical settings, its application to animal training has grown steadily over the past two decades, with particularly promising results for addressing welfare challenges in zoological facilities [5,6]. While this review focuses on zoological institutions to align with the scope of this Special Issue, the constructional principles described are broadly applicable across other professional managed care settings that house animals for conservation, education, research, or welfare purposes, including aquariums, sanctuaries, and rehabilitation centers. The term “zoo animals” is used here to denote animals in such professionally managed programs.

Kaplan-Reimer et al. [7] provides a behavioral definition of compassion particularly relevant to animal care: “Compassionate contexts foster shifts in functional relationships… from aversive to increasingly appetitive” and “Compassionate behavior… is appetitive behavior whose effects create such contexts for the self or for other(s)” (p. 3). By focusing on building behaviors rather than eliminating them, the constructional approach creates precisely these shifts toward more appetitive functional relationships.

The constructional approach represents a compassionate framework for animal behavior change. While compassion has many definitions across disciplines, Scallan and Rosales-Ruiz [8] characterize it by three essential features that are deeply integrated into constructional programs:

1. **Identifying behavior challenges without judgment:** Rather than labeling animals’ behaviors as “problems”, “maladaptive”, or “abnormal”, the constructional approach recognizes that challenging behaviors are often competent responses to difficult circumstances [9]. As Goldiamond [4] noted, “What is considered pathology may also be defined as a competent operant, maintained by the environmental reinforcers it produces, but presently (or foreseeably) producing these at high cost” (p. 158). For zoo animals, this perspective allows caregivers to view stereotypic behaviors, non-compliance, aggression, or fear responses as logical outcomes of specific contingencies rather than as flaws in the animal. Throughout this paper, commonly used descriptive terms such as “aggressive behavior”, “fear”, or “non-compliance” are retained for clarity and accessibility to zoo professionals; however, they are used descriptively rather than judgmentally and are understood within a constructional framework as behaviors maintained by identifiable functional contingencies.
2. **Building empathy:** The constructional approach fosters a deeper understanding of animals’ perspectives by examining the contingencies that maintain behavior. This shifts caregivers from asking “How do we stop this behavior?” to “What does this animal need, and what alternative ways can we provide to meet those needs?” This empathy—understanding that behavior makes sense in context—leads to more effective and less coercive interventions.
3. **Taking action to enhance well-being:** Rather than using extinction, punishment, or other aversive procedures that may temporarily suppress behavior but cause additional distress, the constructional approach actively builds repertoires that give animals access to critical reinforcers through multiple pathways. This proactive stance on enhancing well-being aligns with how compassion is enacted.

This paper aims to introduce the constructional approach as a valuable tool for enhancing zoo animal welfare management by:

1. Outlining the core principles and historical development of the constructional approach
2. Distinguishing it from traditional animal training methodologies
3. Presenting evidence of its effectiveness through case examples
4. Providing practical recommendations for implementation in zoo settings
5. Discussing how the approach aligns with evolving conceptions of animal welfare, including compassionate care practices

## 3. Materials and Methods

This review follows a narrative approach to examine and synthesize literature on the constructional approach and its applications to zoo animal welfare. The methodology involved three phases:

First, a comprehensive search was conducted for peer-reviewed publications on the constructional approach, including foundational texts, articles on the application of the constructional approach to human and animal behavior, and additional papers addressing assent, compassion, and zoo welfare specifically. Databases searched included Google Scholar, Web of Science, and PubMed using the following search terms: “constructional approach”, “animal training”, “zoo animal welfare”, “behavioral contingencies”, “assent”, “compassion”, and “degrees of freedom”.

Second, master’s theses from the University of North Texas, which houses a significant body of research on constructional approaches, were identified and reviewed [10,11,12,13,14].

Third, documented case examples of constructional applications in zoo settings were collected from professionally respected educational and organizational sources, including the European Association of Zoos and Aquaria (EAZA), EAZA Academy, the Animal Behavior Management Alliance, the Animal Training Fundamentals Library, the Association of Behavior Analysis International, and the journal Operants published by the B.F. Skinner Foundation [15].

The collected literature and professional materials were analyzed to identify the principles, techniques, and outcomes of constructional approaches. Particular attention was paid to comparing these with commonly promoted hierarchical models of behavior change procedures. This comparison focused especially on practical applications relevant to zoo animal management.

## 4. Theoretical Framework and Development of the Constructional Approach

### 4.1. Contemporary Context and Historical Origins

This section provides the theoretical foundation for the constructional approach, outlining its historical origins, defining features, and distinguishing principles that inform later applied examples.

The constructional approach, a methodologically rigorous framework, has gained particular relevance in response to recent trends in animal training. Over the past two decades, procedural hierarchies that prescribe intervention sequences without individualized functional assessment have been frequently promoted in animal training contexts. However, these hierarchies risk oversimplifying complex behavioral processes. Farhoody [16] critiques such hierarchies as contradictory to foundational principles of behavior analysis, warning of ineffective and misleading applications. Fernandez [17] similarly emphasizes that ethical and effective behavior change cannot be guided solely by minimizing procedural intrusions, but must be grounded in functional contingency analysis and constructional repertoire building. Procedural hierarchies, such as least-to-most intrusive models, have been criticized for oversimplifying complex decision-making processes and failing to meet individual behavioral needs. Johnston and Sherman [18] described hierarchies as products of efforts to find quick solutions rather than individualized functional analyses. Mace [19] further noted that because such models often misalign treatments with behavioral function, they can lead to abandonment or escalation of ineffective procedures. Fisher et al. [20] demonstrated that rigid adherence to hierarchical progression can delay effective intervention, inadvertently reinforce undesired behavior, and increase exposure to unnecessary or aversive procedures. Constructional approaches provide a scientifically grounded alternative, emphasizing individualized, contingency-based interventions that maximize beneficial outcomes for animals. This represents an important advancement in animal welfare science, as it provides a more ethical and effective framework for addressing behavioral challenges in zoo settings.

The constructional approach originated with Israel Goldiamond’s seminal 1974 paper Toward a Constructional Approach to Social Problems: Ethical and Constitutional Issues Raised by Applied Behavior Analysis, which proposed a fundamental shift in behavioral intervention design [4]. Rather than focusing on eliminating problem behaviors (a “pathological” approach), Goldiamond advocated for building desirable behavioral repertoires (a “constructional” approach) [4,9]. This work was later developed and refined by behavioral scientists, including T.V. Joe Layng, Paul T. Andronis, and Jesús Rosales-Ruiz, who extended its applications across various contexts, including animal behavior management [4,6].

The approach gained traction in animal training contexts, primarily with domestic species, through the work of researchers at the University of North Texas [10,11,12,13,14,21,22,23], private practitioners working with companion animals (dogs and reptiles) [24,25,26], and practitioners who demonstrated its effectiveness with diverse species in zoological settings and wildlife sanctuaries [5,27,28,29,30,31,32,33,34,35,36]. Far from being a recent trend or fad, the constructional approach represents a methodologically rigorous framework grounded in over 50 years of behavioral science that is gaining traction in the zoological community for compelling reasons.

### 4.2. Five Critical Elements of the Constructional Approach

The constructional approach is structured around five critical elements that guide intervention design [4,6,34].

1. **Terminal Repertoire:** Identifying “where you want to go from here”—the ultimate behavioral goal.
2. **Current Relevant Repertoire:** Assessing “where you are now”—the existing behavioral skills and patterns.
3. **Change Procedures:** Determining “how you can get there”—the specific training techniques to be employed.
4. **Maintaining Consequences:** Establishing “what would keep you going”—the natural reinforcers that will sustain the behavior.
5. **Progress Monitoring:** Deciding “how you will know where you are along the way”—the metrics for evaluating success.

These elements form a comprehensive framework that extends beyond a simple procedural checklist to encompass a holistic approach to behavior change and have been applied with animals in zoological settings [34].

### 4.3. Key Distinguishing Features

The constructional approach is not defined by a single procedure, but by a system of interlocking features that shift how behavior is understood, analyzed, and shaped. What distinguishes this approach from traditional animal training is its emphasis on building behavior rather than eliminating it, using analysis tools that move beyond simple cause-and-effect models [4,6]. This section outlines the key distinguishing features of the constructional approach, beginning with its foundation in repertoire-building, followed by its reliance on Nonlinear Contingency Analysis (NCA), and its principled use of reinforcement based on functional assessment. From there, we explore how constructional programs promote meaningful choice through degrees of freedom, how emotional behaviors are viewed through the lens of contingency analysis, and how these principles support a compassionate framework for both animals and caregivers. Each of these elements is described in greater detail in the sections that follow, providing a clear picture of how constructional programs produce sustainable, welfare-centered outcomes.

#### 4.3.1. Building vs. Eliminating Behavior

A fundamental principle of the constructional approach is its focus on building desirable behaviors rather than eliminating problematic ones. Instead of asking “How can we stop this undesirable behavior?” the constructional caregiver asks, “What behavior is missing from this animal’s repertoire that would make the undesirable behavior unnecessary?” [4]. This shift in focus avoids the potential welfare concerns associated with behavior reduction procedures [37].

#### 4.3.2. Nonlinear Contingency Analysis (NCA)

The constructional approach utilizes a sophisticated analytical framework called Nonlinear Contingency Analysis (NCA), which examines the complex web of contingencies maintaining a behavior, including:

- Multiple environmental factors
- Competing contingencies
- Costs and benefits of different behavioral options
- Emotional components of behavioral patterns

This systemic analysis goes beyond the linear A-B-C (Antecedent-Behavior-Consequence) model commonly used in traditional approaches, enabling more effective and welfare-centered interventions [4,5,6].

The critical feature of NCA is that it considers multiple interacting contingencies, allowing practitioners to identify when one contingency has been superimposed on another. NCA helps address the underlying contingency. For instance, when animals display fear or aggression toward caregivers, these behaviors may be maintained by negative reinforcement; the animal’s behavior successfully creates distance from an aversive stimulus, such as the caregiver. Traditional approaches often attempt to address this by superimposing a positive reinforcement contingency, such as training the animal to sit on a station for food while a caregiver is present. While this may appear successful on the surface, the fear or aggression contingency remains unaddressed. As Pedersen [33] explains, these strategies often fail to contact the contingency maintaining the original behavior; rather than resolving it, they layer a new behavior on top, reinforced by food.

When a contingency is merely superimposed rather than addressed, the original behavior often reappears when the superimposed contingency is no longer maintained. For example, if a bird is trained to sit on a perch for food while a feared caregiver is present, and the caregiver stops reinforcing this behavior, the original fear/aggression response will likely return.

NCA helps caregivers identify superimposed contingencies and develop interventions that directly address the critical consequences (functional reinforcers) maintaining the behavior. This results in more durable behavior change and improved welfare because the root causes of the behavior are addressed rather than merely masked [37].

#### 4.3.3. Appropriate Application of Reinforcement

The constructional approach employs both positive reinforcement and negative reinforcement but does so based on a careful assessment of existing contingencies rather than an arbitrary preference for one procedure over another. Importantly, it avoids procedures like punishment, extinction, differential reinforcement of incompatible/alternative behaviors (DRI/DRA), and systematic desensitization/counter-conditioning, which can produce emotional side effects, welfare concerns, and often unsustainable results [28].

When an animal’s behavior is maintained by negative reinforcement, such as when animals move away from people, objects, or specific conditions, the constructional approach recognizes this as valuable information about what is reinforcing for the animal. Rather than attempting to override this contingency with positive reinforcement, constructional caregivers identify the critical consequence (distance or removal of aversive stimuli) and use it strategically to shape new desired responses. Heidenreich [28] emphasizes that understanding how to shape behaviors from fear or aggression to calm by providing distance can transform animals. Animals learn to use their behavior to control their outcomes.

This distinction is particularly important because in zoological settings, caregivers are not purposely inserting something unpleasant into the environment to occasion behavior. Rather, these stimuli are either already present in the environment or may need to be present for desired goal behaviors, such as participation in medical care. By developing interventions that use the existing functional reinforcer (which may be distance) appropriately, animals can learn new, more adaptive ways to achieve the same reinforcing outcome.

#### 4.3.4. Degrees of Freedom and Assent

The constructional approach emphasizes providing animals with genuine choice through increasing degrees of freedom. As described by Goldiamond [38,39] and as cited in Heidenreich and Layng [15], genuine choice occurs only when an individual has more than one way to obtain a critical consequence. If an animal has only one pathway to a critical reinforcer, participation becomes effectively coerced.

This is captured mathematically in the concept of degrees of freedom (n−1), which clarifies that when only one path to a critical consequence exists, degrees of freedom equal zero [15,38,40]. Will and Nishimuta [41] further emphasize that for genuine choice to exist, all elements of the contingency, occasion, behavior, and consequence must be equally available for two or more options. In other words, the animal must be able to emit alternative behaviors and access the same reinforcer through multiple functional pathways, and the occasioning stimulus conditions must be available.

It is important to distinguish genuine choice from interpretations of “control” commonly used outside behavior analysis. As Farhoody [16] emphasizes, terms like “choice” and “control” are often used as explanatory fictions that obscure the functional relations between environmental events and emitted behavior. In behavior analysis, control refers to the observable relationship between environmental conditions and the behaviors they occasion and maintain, not an internal possession or motivation. When animals emit behaviors often labeled as “control seeking”, such as moving away from aversive conditions or toward preferred stimuli, it is the access to meaningful consequences, not an intrinsic need for control, that maintains the behavior. For example, the option to move under a tree may be highly reinforcing on a hot day but have little relevance when temperatures are mild. Thus, behavior is shaped by environmental conditions and the availability of meaningful outcomes.

Goldiamond’s framework offers a more scientifically grounded view of what is often mischaracterized as control. Providing multiple functional pathways to critical consequences reduces coercion and supports animals in selecting behaviors based on activity-specific consequences, such as differences in effort, comfort, distance from aversive stimuli, or proximity to preferred stimuli [15].

McGee’s [22] research with rats highlights the practical importance of analyzing degrees of freedom when assessing assent. In her study, rats initially appeared to be emitting a focal behavior to access food. However, further analysis revealed that their behavior was primarily maintained by escape from the training context. This became evident only when the rats were provided with multiple ways to access both outcomes: food could be obtained by completing a trained task or via free feeding, and escape could be achieved either by entering a tunnel or by leaving the training platform. The rats reliably chose escape, indicating that the critical consequence maintaining their behavior was not food, but removal from the stimulus conditions. This finding illustrates how behavior may appear cooperative, but without increased degrees of freedom for all relevant consequences, participation may be maintained by escape rather than by engagement. This distinction parallels earlier examples where a positive reinforcement contingency was superimposed without contacting the original contingency maintaining undesired behavior.

In applied settings, assent refers to an animal’s ongoing agreement to participate, distinct from consent, which is provided before an event begins [42]. Genuine assent requires uncoerced participation, which can only be assessed when animals have meaningful, functional alternatives available.

Although the concept of assent is well established within constructional and compassionate frameworks, methods for its systematic evaluation continue to evolve. Ongoing refinement of how assent is analyzed and measured across contexts will further strengthen both its conceptual clarity and its practical application within animal training and welfare research.

In applied terms, caregivers should ensure that animals are familiar with and able to emit the behaviors available to them under the current conditions.

For trainers and animal caregivers, this perspective has practical implications:

1. Identify the critical consequences maintaining the animal’s responding.
2. Ensure the behaviors available to the animal are within its learned repertoire and can be emitted under the current conditions.
3. Offer multiple behaviors that can access the same reinforcing outcome.
4. Verify that the available options provide genuinely equivalent reinforcing outcomes under current conditions.
5. The topography of the behavior (what the behavior looks like) does not provide enough information to indicate coercion or cooperation. Giving the animal genuine alternative options provides more information.

By thoughtfully arranging degrees of freedom and assessing assent based on functional contingencies rather than appearance, caregivers can promote what Goldiamond described as behavioral freedom: a continuum reflecting the number of available, meaningful pathways to critical outcomes. While Skinner emphasized that freedom was not an internal state but the observable absence of aversive control, Goldiamond elaborated that freedom increases as degrees of choice expand. Even positive reinforcement can be coercive when only one behavioral option leads to reinforcement. Providing multiple functional pathways to important consequences reduces coercion, supports sustained participation, and enhances the animal’s ability to interact effectively with its environment under conditions that promote well-being.

##### Constructional Approaches to Addressing Fear Responses

Incorporating degrees of freedom has effectively addressed fear (and aggression), representing a measurable advancement over traditional approaches. Conventional fear treatment methods, such as habituation, systematic desensitization, and counter conditioning, typically rely on stopping or reducing unwanted responses via the extinction of fear responses. As Layng and Abdel-Jalil [43] explain, these approaches attempt to eliminate fearful reactions by gradually exposing animals to frightening stimuli, often producing slow, incremental progress with questionable long-term results. These methods fundamentally focus on eliminating problem behaviors rather than building alternative responses. This reduction-focused orientation frequently leads to animal distress and treatment failure.

In contrast, Constructional Exposure Therapy (CET) represents a fundamentally different approach by leveraging Goldiamond’s degrees of freedom model. CET ensures animals always have at least one degree of freedom by providing two ways to achieve the same important outcome, distance from aversive stimuli: (1) remaining calm, or (2) showing distress. This means the animal is “never asked to give up a consequence important to them, providing genuine choice and some control over the situation” [44] (p. 10). Instead of making the animal “get used to” frightening stimuli or forcing them to ignore their fear, CET works with what’s already reinforcing for the animal, distance, by making it available for a wide variety of progressively calmer behaviors.

The results of this constructional approach are measurable both in speed and effectiveness. Animals previously considered untrainable show an observable transformation of fearful and aggressive responses into calm, cooperative behavior, often with minimal sessions and “little distress experienced” [43] (p. 10). The de Fernandes and Dittrich [40] paper further supports this application of Goldiamond’s framework, showing how increasing genuine choice creates more lasting behavior change by building desirable alternative responses rather than merely suppressing unwanted behaviors. This fundamental distinction, building rather than eliminating, represents the essential contrast between constructional and traditional approaches to animal training.

#### 4.3.5. Emotions as Contingency Descriptors

Rather than treating emotions as internal causes of behavior, the constructional approach views emotions as the descriptors of the relationships between contingencies [4,9,39]. Emotions are not the consequences themselves but rather describe the functional relationships between behavior and its maintaining contingencies. As Layng [45] explains, “changes in emotion track changes in consequential contingencies. That is, emotions reflect the contingency requirements we face. As these requirements change, so do our emotions” (p. 6).

It is important to distinguish between emotions and emotional behaviors. Emotional behaviors are the observable responses, such as approach/avoidance patterns, body postures, vocalizations, and physiological changes, that can be directly measured and analyzed. Emotions, in contrast, describe the contingency relationships that occasion and maintain these observable behaviors. This distinction is critical because it shifts focus from attempting to change internal emotional states to modifying the environmental contingencies that produce observable emotional behaviors. See Table 1.

This perspective transforms how we understand and address emotional responses. When an animal displays behaviors commonly labeled as fear, frustration, or aggression, constructional caregivers focus on altering the underlying contingencies rather than attempting to directly counter-condition emotional responses. For example, when a black bear displays aggressive jaw-popping behavior in the presence of multiple people, this behavior describes a distancing contingency; the animal is attempting to increase distance from what it perceives as aversive. By recognizing this contingency and working with it rather than against it, caregivers can shape alternative calm emotional behaviors that produce the same functional reinforcer (distance), gradually transforming the emotional behavior pattern.

This approach echoes early work by Skinner [46,47], who emphasized that emotions are not causes but rather part of the functional relations shaped by environmental contingencies. By addressing these contingencies rather than attempting to manipulate emotions directly, constructional caregivers can achieve rapid, measurable improvements in observable emotional behaviors and the welfare outcomes they reflect.

This framework aligns with contemporary understanding of emotions as important welfare indicators and can provide clear, measurable pathways for improving emotional welfare through contingency management rather than through attempts to directly suppress or counter-condition emotional responses.

## 5. Applications of the Constructional Approach in Zoo Settings

### 5.1. Research Evidence

Building on the preceding theoretical discussion, this section illustrates how those principles are applied in zoological practice through documented examples and case studies.

The effectiveness of constructional techniques has been demonstrated through several empirical studies with relevance to zoo contexts:

- Katz and Rosales-Ruiz [21] utilized a constructional approach to successfully address behaviors commonly labeled as fear in shelter dogs, demonstrating a method for teaching animals that displayed avoidance responses to approach and interact with novel people.
- Fernandez [14] employed negative reinforcement shaping procedures to transform petting zoo sheep that exhibited behaviors typically described as fearful into animals that would approach and allow contact from humans. This research demonstrated that recognizing and working with the functional reinforcers maintaining behavior (distance from humans) provided a foundation for establishing more affiliative human–animal interactions.
- McGee’s [22] research shows that participation in positive reinforcement-based teaching may still be coerced if no alternative ways to access reinforcement are available. By increasing degrees of freedom and offering meaningful alternatives, critical consequences can be revealed, and caregivers can better assess whether an animal’s participation reflects genuine assent.
- Snider [10] developed a Constructional Canine Aggression Treatment that uses shaping with negative reinforcement to effectively increase desired responses without the use of punishment or extinction.
- Rentfro [11] adapted similar methodologies for domestic cats, creating what was termed a “Fearful to Friendly” protocol with applications for felids in zoological collections.
- Ward [13] and Hardaway [12] demonstrated constructional approaches to husbandry behaviors in equids, with potential applications for equid species in zoo settings.

These studies offer empirical support for constructional methods across diverse species and settings within the zoo and aquarium community.

### 5.2. Practical Applications in Zoo Settings

EAZA acknowledges that the application of evidence-based training technologies plays a crucial role in promoting optimal animal well-being in managed care environments. As a result, training is considered an essential component of modern zoological care practices [48]. EAZA further highlights a broad range of beneficial outcomes that well-designed training programs can produce, including cooperation in medical care, participation in daily husbandry, transformation of undesired behavioral patterns, facilitation of data collection for scientific research, enhancement of human health and safety protocols, enrichment of the animal’s experience, support for rehabilitation and conservation efforts, and participation in educational initiatives.

While these outcomes are well-documented, achieving them can require addressing complex behavioral challenges. The constructional approach builds on familiar scientific foundations but introduces key distinctions previously discussed, such as genuine choice, NCA, and a focus on building rather than reducing behavior, which make it more effective in addressing complex challenges [4,6]. It provides practical solutions to behavior challenges that traditional models have often struggled to resolve and enables the achievement of previously unattainable training outcomes.

The following examples and case studies illustrate how the various components of the constructional approach are incorporated into behavior management and successfully applied in zoological settings. They demonstrate their value in achieving sustainable behavior change and enhancing the welfare of a diverse range of species.

#### 5.2.1. Medical and Husbandry Training

Constructional methods have been particularly valuable for establishing medical and husbandry behaviors that maintain genuine assent throughout the process. By analyzing the contingencies maintaining avoidance behaviors and applying appropriate shaping procedures, animals can develop repertoires that enable their participation with assent in their care without the use of restraint or other aversive techniques.

**Domestic Rabbits (*Oryctolagus cuniculus domesticus*): Successfully Reading Microchips**

As shared by Pedersen [33] a group of identical-looking rabbits at the Copenhagen Zoo in Copenhagen, Denmark, needed to be individually identified through microchips, but they showed escape and avoidance responses to the microchip reader. Traditional approaches might have involved restraining the rabbits or attempting counter-conditioning with food.

Instead, a constructional approach was implemented, focusing on the critical consequence maintaining the fear responses, distance from the microchip reader. The intervention began by presenting the reader at a distance where the rabbits could emit calm emotional behaviors, then immediately removing it, reinforcing their calm responses with the distance they desired.

With the rabbits emitting relaxed behavior in the presence of the reader, the caregiver gradually decreased distance while continuing to reinforce calm responses by withdrawing the device. Eventually, the rabbits not only tolerated the microchip reader but actually approached it, allowing the caregiver to transition to positive reinforcement using preferred foods (vegetables and pellets) when the rabbits touched or remained near the reader.

This case demonstrates how addressing the functional reinforcer (distance) provided a foundation upon which positive reinforcement could be effectively built. By creating multiple degrees of freedom, rabbits could either run away or emit various calm responses to achieve distance from the aversive stimulus; the animals could give genuine assent to a previously fear-evoking procedure.

**Indian Rhinoceros (*Rhinoceros unicornis*): Building a Better Lean-In Behavior**

Heidenreich and Layng [15] describe a case with a female Indian rhinoceros at Fota Wildlife Park in Cork, Ireland. Caregivers were attempting to shape the animal’s “lean in” behavior for medical examinations. This behavior, where the animal presses its side against a perimeter barrier, allows caregivers safe access to body parts for medical procedures. The female had an older calf housed in a nearby enclosure, visible during training sessions.

The team observed that the female often left the session to visit her calf or offered other behaviors for which she had reinforcement history, specifically “open mouth” behaviors and “foot presentation”. Rather than viewing these as distractions or non-compliance, the team recognized these behaviors as valuable information indicating that reinforcement for the “lean in” behavior was insufficient.

By increasing the degrees of freedom, reinforcing instances of “open mouth” behaviors, “foot presentation”, and smaller approximations of the “lean in” response, the caregivers successfully built the desired behavior. As the team developed reinforcement history for multiple behaviors, they observed that the “open mouth” and “foot presentation” behaviors were emitted less frequently, while the “lean in” response increased.

Notably, once degrees of freedom were increased, the rhinoceros showed no interest in disengaging from the session to visit her calf, demonstrating how this approach can simultaneously enhance training effectiveness and animal welfare. This case illustrates how recognizing and utilizing degrees of freedom provides critical information that helps caregivers achieve desired outcomes more efficiently.

**Jaguar (*Panthera onca*): Offering Choice During Injection Training**

An innovative application of degrees of freedom involved creating options for injection sites with a jaguar at the Audubon Zoo in New Orleans, Louisiana, United States [29]. While many zoological training programs teach animals to present specific body parts on cue (right ear, left ear, right hip, left hip), this case implemented a special cue meaning “it’s your choice”.

This cue allowed the jaguar to choose which body part to present for injections, increasing the animal’s agency by providing a degree of freedom for a potentially aversive procedure. This approach is particularly valuable for situations where animals might have preferences or sensitivities about where medical procedures are performed, creating a system where the animal can communicate those preferences while still participating in necessary care.

#### 5.2.2. Creating Communication Pathways

The constructional approach emphasizes establishing ways for animals to communicate preferences or withdraw assent, particularly in situations where traditional training would offer only compliance or refusal.

**Amur Leopard (*Panthera pardus orientalis*): Using a Bell to Communicate Preferences**

In an example shared by Heidenreich [29], an Amur leopard at the Santa Barbara Zoo in Santa Barbara, CA, USA, was being trained for an open-mouth behavior when the animal began ringing a bell that already had reinforcement history. While caregivers did not reinforce the bell-ringing during that particular session, they recognized its potential as a means of communication.

The team later discussed how they could reinforce the bell-ringing as a way for the leopard to communicate “I’m not sure what I’m supposed to do” or “I would prefer to do this behavior”. This would serve as a way for the leopard to “say no” to a requested behavior while still receiving reinforcement, thereby maintaining a successful training relationship that honors the animal’s preferences.

This case illustrates how constructional programs identify and build upon behaviors the animal already offers or can learn as potential communication pathways, rather than insisting on a single “correct” response. By allowing the leopard multiple ways to receive reinforcement, including a way to decline participation, caregivers created a system with increased degrees of freedom that enhanced welfare while still achieving training goals. The bell is now present for all training and provides the leopard with a behavioral alternative to communicate and gain reinforcement, while also giving caregivers information to make adjustments that facilitate attaining desired goals.

**Giraffe (*Giraffa reticulata*): Building Touch Behaviors Through Alternative Options**

Another example provided by Heidenreich [29] involved a female reticulated giraffe in a private collection located in Texas, United States, who was not receptive to tactile stimulation and was being trained for a touch behavior that would ultimately support medical care. Rather than focusing exclusively on the desired behavior using traditional methods such as systematic desensitization and counter conditioning, caregivers created a constructional environment by providing readily available feeding devices throughout the habitat that delivered the exact grain being offered by the caregiver.

This arrangement allowed the giraffe to engage with the caregiver or access food independently from the devices. When working on teaching the giraffe to bring her head to the caregiver’s hand, she always had the option to go to the feeding device instead if anything about the training was aversive, confusing, or too difficult.

This system allowed the giraffe to effectively communicate “no” to the touch behavior while still receiving reinforcement. This compassionate approach to training assured the giraffe did not have to miss out on reinforcement if conditions or expectations exceeded her abilities at that time. The information gathered from these choices was integral to refining the program. She typically opted out when the male giraffe moved too close; while she preferred his companionship, she also required that he maintain some distance. Opting out was also more likely when tactile contact was initiated by the caregiver rather than when the caregiver’s hand remained still and she was prompted to move toward it. Initial approximations began near specific body parts, such as close to the mouth, and gradually expanded to other areas of the head. Attempts to start at other locations often lead to opting out. These observations provided valuable feedback for adjusting the procedure to maintain the animal’s assent and comfort. The strategy proved efficient and effective, with the giraffe rarely opting to feed from the easily accessed nearby devices. More importantly, the previously observed kicking behavior diminished from her repertoire, and the touch behavior was trained rapidly with the system in place.

#### 5.2.3. Addressing Challenging Environmental Conditions

Animals in zoological settings can experience environmental conditions that function as aversive stimuli, occasioning fear or avoidance responses. Rather than attempting to suppress these responses, the constructional approach focuses on identifying the critical consequences that maintain them and building new, desirable response patterns. By offering degrees of freedom, multiple ways for the animal to access meaningful outcomes, animals are given opportunities to navigate challenging conditions successfully. This strategy not only addresses the environmental challenges but also supports the development of more sustainable and preferred behavioral repertoires. The following examples illustrate how these principles are applied in practice.

**Gorilla (*Gorilla gorilla*): Successful Door Management**

A case study presented by Heidenreich [49] at the EAZA Welfare Plenary featured the lowland gorilla at the Audubon Zoo in New Orleans, LA, USA, who had been successfully trained to station near caregivers for positive reinforcement. However, challenges arose during door management procedures in the back habitat area. When staff attempted to close doors, the gorilla, along with three other individuals, emitted escape and avoidance behaviors. These behaviors, shaped by past experiences with door closure, made routine habitat management more complex.

Previous strategies, including offering food while gradually attempting to close the doors (consistent with systematic desensitization and counterconditioning practices), were implemented thoughtfully but yielded limited success over several months. Further contingency analysis revealed that access to food was not the critical consequence maintaining the gorillas’ behavior. Instead, the opportunity to move away and prevent door closure functioned as the critical consequence (negative reinforcement). Remaining stationary had no influence on the door’s movement, making leaving the area the more effective behavior from the animals’ perspective.

The team implemented a procedure directly targeting the critical contingency to address this. Rather than making food contingent on stationing, they rearranged contingencies so that door movement itself was responsive to the gorilla’s behavior. When the gorilla remained in position, the door handle was briefly touched but immediately returned to the open position. The gorilla learned that many calm emotional behaviors could maintain the door’s open status through this arrangement.

Within a single session, the gorilla learned that calm behaviors, especially those offered at a station, effectively influenced the door’s position. By focusing on the functional reinforcer, control over the door’s movement, rather than relying on delivering food items that did not address the animal’s critical consequence, caregivers achieved rapid progress in resolving a longstanding training challenge. The team soon successfully closed the door with the gorilla calmly seated, demonstrating continued participation under conditions arranged to maintain meaningful choice and access to critical outcomes.

This approach exemplified compassion in three critical ways. First, it recognized the gorillas’ behaviors as competent responses to prior environmental contingencies, without judgment. Second, it fostered understanding by analyzing what meaningful outcomes were maintaining the animals’ responding, shifting the focus from “How do we stop avoidance?” to “What does this animal need, and how can we meet those needs?” Third, it took action to enhance well-being by constructing multiple pathways to critical consequences, rather than relying on extinction or punishment-based methods.

By increasing degrees of freedom, the procedure allowed various calm responses to maintain the door’s open status, reducing coercion and preserving previously effective behaviors (moving towards the door) without extinguishing them. This strategy minimized the risk of frustration or escalation that can occur when effective behaviors are abruptly rendered ineffective.

These procedures were successfully implemented across all four gorillas. All individuals learned to remain calm in the back area during habitat servicing with door closure, reflecting constructional success based on genuine assent and improved welfare. This case illustrates how interventions designed around functional relationships can rapidly and sustainably build new behavioral repertoires, even in contexts that had previously presented persistent challenges.

**Camel (*Camelus bactrianus*): Rebuilding Scale Training**

Pedersen [33] shared an example with a camel at the Copenhagen Zoo in Copenhagen, Denmark, who had been trained to step onto a scale, but after experiencing the scale move underneath her feet during a weighing session, she began emitting a strong avoidance behavior. Initial attempts using positive reinforcement with carrots and systematic desensitization failed, as evidenced by the camel’s emotional behaviors, which included tail swishing, leaning backward, refusing to chew, and even tripping over barriers to avoid the scale.

NCA revealed that traditional methods were attempting to superimpose a positive reinforcement contingency (carrots for approaching the scale) on top of a negative reinforcement contingency (avoiding the scale), without addressing the critical consequence maintaining the avoidance behavior.

The team shifted to a constructional approach, having the camel approach the scale only to the point where she remained calm. Then, immediately, the team asked her to move away, providing the distance she sought. Food was used only as a positioning tool, not contingent on engaging with the scale. By reinforcing calm emotional behaviors with the distance the camel wanted, the team built a new pattern of behavior.

Within a single session, the camel transformed from refusing to approach the scale to comfortably standing on it with all four feet. Her emotional behaviors changed, showing relaxation rather than tension. This case illustrates how identifying and working with the functional reinforcer rather than against it can achieve rapid behavior change while maintaining genuine assent throughout the process.

This case exemplifies compassion through all three key elements: First, it identified the camel’s avoidance behavior not as stubbornness but as a competent response to a frightening experience (identifying behavior challenges without judgment). Second, it built empathy by analyzing what mattered to the camel, distance from the scale, rather than imposing human expectations about what “should” motivate her. Third, it took action to enhance well-being by creating a pathway where the camel could gradually build behaviors with genuine choice, rather than forcing compliance through systematic desensitization or counter conditioning procedures that would have caused additional distress.

**Sheep (*Ovis aries*): Transforming Hesitation to Approach People**

In this case study from Heidenreich [5] with a domestic sheep at Sequoia Park Zoo, located in Eureka, CA, USA, the animal was hesitant to approach caregivers to take food, jumping through a small window opening in the wall to return to a space with two preferred companion goats when a caregiver approached.

A NCA revealed multiple contingencies operating simultaneously rather than a simple linear relationship. While food functioned as an appetitive stimulus (positive reinforcement contingency), the analysis identified several competing negative reinforcement contingencies: the small, enclosed space, separation from goats, and proximity to people all functioned as aversive conditions the sheep was trying to avoid. Traditional approaches often focus only on the linear food-behavior relationship while overlooking these competing contingencies, which explains why simply offering higher-value food items often fails.

This comprehensive analysis exemplifies the first element of compassion described by Scallan and Rosales-Ruiz [8]: identifying behavior challenges without judgment. Rather than labeling the sheep as “shy”, “stubborn”, or “difficult”, caregivers recognized its behavior as a competent response to environmental contingencies, a way of accessing critical reinforcers (social contact with goats) and avoiding perceived threats (isolation, confinement, human proximity).

Rather than attempting to counter-condition the behavior by offering increasingly valued food items (superimposing a positive reinforcement contingency), the intervention directly addressed the negative reinforcement contingencies. Caregivers opened the door separating the two stalls, allowing the sheep to have the distance she desired from people, and brought the goats into the space where the caregiver was.

This approach demonstrates the second and third elements of compassion: building empathy by understanding the contingencies from the sheep’s perspective, and taking action to enhance well-being by providing multiple pathways to critical reinforcers. By recognizing and working with the functional reinforcers maintaining the sheep’s behavior (access to goats, avoidance of confinement), the intervention created conditions where positive reinforcement could be effective without competing with negative reinforcement.

This compassionate application of NCA resulted in the sheep comfortably taking food from the caregiver’s hand in less than twenty minutes, a transformation that traditional methods focused only on positive reinforcement had failed to achieve despite extended efforts. This case illustrates how addressing all relevant contingencies, rather than focusing on a single linear relationship, creates more effective and welfare-enhancing outcomes.

### 5.3. Addressing Fear and Aggressive Behavior

The constructional approach offers effective alternatives to traditional methods for addressing fear and aggression in zoo animals through the strategic use of negative reinforcement with increased degrees of freedom.

Animals regularly demonstrate fear or aggressive responses that are maintained by negative reinforcement, the removal of or distance from something the animal perceives as aversive. As Heidenreich [28] notes, animals who cannot remove themselves from the stimulus may emit aggressive responses to drive things away. This response can include growling, lunging, biting, or charging toward the habitat boundary to move people or other animals away. Rather than attempting to suppress these responses through counter-conditioning or punishment, the constructional approach recognizes them as functional behaviors that provide information about what reinforcers are operating [37].

#### 5.3.1. Key Principles in Addressing Fear and Aggressive Responses

A key principle in addressing these behaviors is to begin under conditions where the animal is likely to succeed, meaning conditions where desired responses are readily emitted. This typically involves arranging the aversive stimulus at a sufficient distance so that the animal emits relaxed emotional behaviors. Under these favorable conditions, caregivers can shape new behavioral repertoires that produce the same reinforcing outcome the animal seeks (often access to increased distance). By gradually changing criteria, such as moving closer, staying longer, or otherwise changing the stimulus conditions, while reinforcing calm emotional behaviors with distance, caregivers can transform fear and aggressive responses into approach and cooperation.

Importantly, while traditional training approaches often rely on procedures such as extinction, differential reinforcement of alternative behaviors (DRA), or differential reinforcement of incompatible behaviors (DRI), constructional interventions minimize the need for these strategies by arranging initial conditions that support the emission of desired behavioral responses. When stimulus conditions are structured to occasion calm, functional responding, caregivers avoid creating scenarios where extinction would otherwise be considered necessary. Even if undesired responses occasionally emerge, access to reinforcement is preserved rather than withheld. Instead of reinforcing some behaviors while withholding reinforcement for others, as in DRA or DRI, the constructional approach ensures that reinforcement remains continuously available across multiple behavioral options. This strategy reduces coercive conditions by increasing the animal’s degrees of freedom, supporting sustained participation in ways that are more aligned with the animal’s ability to access meaningful outcomes.

#### 5.3.2. Transforming Fear and Aggressive Responses

The application of these constructional principles to real-world training contexts has revealed notable transformations across taxa and behavioral challenges. The following case studies illustrate how fear and aggressive responses, often considered intractable or requiring aversive control, can be effectively addressed by analyzing the functional contingencies maintaining behavior and arranging conditions that promote calm emotional responding. Each example demonstrates how constructional procedures extend beyond traditional desensitization or counter-conditioning approaches by emphasizing the reinforcement of desired responses under conditions that preserve the animal’s control via genuine choice. Together, these cases showcase how the constructional approach enables practitioners to move toward proactive welfare enhancement.

**Black Bear (*Ursus americanus*): Transforming Undesired Responses Towards People**

Heidenreich and Layng [15] document an example involving a black bear at OC Zoo located in Orange, CA, USA, that would pop her jaw and slap her paw on the ground when two or more people entered her off-display area, resulting in staff leaving. This behavior, maintained by negative reinforcement, presented a challenge for veterinary care. Rather than using conventional counter-conditioning techniques, caregivers implemented a degrees-of-freedom approach where multiple behaviors could produce the same critical consequence of staff distance.

The caregivers observed the bear from a distance where she remained calm, then gradually approached while reinforcing various calm emotional behaviors, such as visual attention, small shifts in body weight, changes in ear position, and remaining stationary by backing away. Within twenty minutes, two people were able to remain close to the bear with the animal emitting desired behaviors. This procedure was then transitioned to positive reinforcement, establishing a foundation for medical care with genuine assent in a previously problematic space. Using a constructional approach-based intervention made it possible to begin training the bear with positive reinforcement to receive essential cold laser therapy for a medical condition.

The compassionate framework is evident in this intervention through recognizing the bear’s jaw-popping and paw-slapping as functional behaviors rather than problematic aggression (identifying challenging behaviors without judgment), understanding the contingencies from the bear’s perspective (empathy), and creating an environment where the bear could build new responses that met her needs while also enabling necessary medical care (taking action to enhance well-being). This approach prioritized the bear’s welfare throughout the process rather than simply achieving compliance.

**Parrot Flock: Transforming Fear Responses Towards Caregivers**

Pedersen [33] documents the experiences with a newly acquired flock of ten parrots, comprised macaws and Amazon parrots (*Ara rubrogenys*, *Amazona brasilliensis*, and *Amazona leucocephala*) at the Copenhagen Zoo, in Copenhagen, Denmark. The birds learned to emit extreme fear responses toward caregivers after being captured with a net and restrained for mandatory annual avian influenza vaccinations. Five to six months after the procedure, the birds continued to emit elevated fear responses, including rapid pacing on perches and alarm calls whenever caregivers approached their aviary. The birds would never enter their indoor area if people were present, limiting care options.

The intervention used a negative reinforcement procedure where a caregiver approached the aviary only to the point where the birds emitted calm emotional behaviors, then immediately retreated, providing the critical consequence (distance) that was maintaining their fear responses. The caregiver carefully monitored emotional behaviors, avoiding sudden movements and being careful not to switch to luring with food, which would superimpose a different contingency.

After several sessions focusing on the birds emitting the most elevated fear responses, the birds began showing curiosity rather than fear responses, gradually moving lower in the aviary. When the birds began approaching on their own, the caregiver introduced food while continuing to observe for any behavioral indicators of the birds’ need for distance.

Within approximately 10–12 sessions, the behavior of all ten parrots, including the Amazon parrots who were not directly worked with, transformed from extreme fear responses to approaching caregivers for food. The success has expanded to recall training and crate training preparation, with the goal of eliminating the need for future restraint during medical procedures. Throughout the process, multiple degrees of freedom were provided, with various feeding stations made available, ensuring that the parrots were never coerced into approaching caregivers for their only access to food.

This case demonstrates how addressing the critical consequence maintaining a well-established fear response can transform behavior across an entire group, and how carefully analyzing contingencies allows for effective intervention without creating emotional conflict.

**Gemsbok (*Oryx gazella*): Transforming Fear Responses Towards People**

Training herds composed of individuals with limited histories of favorable interactions with humans presents unique challenges, particularly when behavior patterns are shaped by contingencies maintaining distance from perceived threats. Heidenreich [27] describes working with a herd of Gemsbok at Sharjah Safari, located in Sharjah, United Arab Emirates, that consistently moved away when caregivers approached the perimeter of their expansive habitat, a clear distancing contingency maintained by the opportunity to increase space from humans.

Rather than attempting to counter-condition this behavior through the delivery of food items (which would have superimposed a new contingency and was not viable given the absence of approach or eating behaviors in the presence of people), caregivers employed a constructional strategy based on NCA. The team established a routine where they would:

1. Stand well outside the habitat boundary and remain stationary at a distance where animals exhibited vigilance but no withdrawal behaviors.
2. When any individual displayed behaviors indicative of decreased vigilance (such as lowering the head, relaxing the ears, or shifting weight), the caregiver would take a step back, reinforcing calmer emotional behaviors with additional distance.
3. This process was repeated, shaping a wide repertoire of progressively calmer behaviors in the presence of humans.
4. Throughout the process, animals consistently had access to an adjacent yard, providing an additional opportunity to increase distance from caregivers if desired, thereby ensuring that escape remained a viable and meaningful behavioral option.

A key insight was recognizing that offering food prior to addressing the existing distance-maintained contingency would have been ineffective. By contacting and working with the critical consequence maintaining behavior (distance), caregivers were able to establish a foundation of calm emotional behaviors that could later be expanded with positive reinforcement procedures.

A unique challenge in herd settings involves the social facilitation of responses: movement from one animal can occasion similar movement from others. Constructional solutions focused on reinforcing calm responses across multiple individuals, both those initially more sensitive to human presence and those demonstrating earlier approximations of comfort. This required an observant, flexible shaping process, where caregivers responded dynamically to behaviors emitted by different individuals across the group. Over time, the combination of shaped individual behaviors produced a cumulative shift in the herd’s collective response.

NCA played a critical role in this success, recognizing that the behavior of each animal served as a contingency influencing the behavior of others. By applying these principles, caregivers shifted conditions to arrange for greater degrees of freedom and genuine assent, resulting in animals that consistently emitted calm emotional behaviors in proximity to humans, resulting in animals approaching humans and eating food. This transformation was achieved without the use of aversive procedures or compulsion, which could increase distress, demonstrating that addressing functional reinforcers directly can produce sustainable improvements in behavior across a range of species.

**Tiger (*Panthera tigris altaica*): Transforming Aggressive Responses Towards a Specific Caregiver**

Pedersen [33] documented a case study at Copenhagen Zoo, in Copenhagen, Denmark, involving a male tiger who displayed aggressive behavior toward one specific caregiver. While the tiger had successfully learned to interact with other staff through conventional desensitization and counter conditioning methods, where caregivers would gradually approach the animal while offering food, this tactic failed with this individual for no identifiable reason. Despite attempts to gradually introduce this person while another caregiver provided preferred food items, the tiger continued to display clear signs of aggression: growling, staring, and maintaining vigilance even while eating.

Pedersen noted that this traditional approach faced a fundamental problem: it relied on the extinction of aggressive behavior while simultaneously superimposing a competing contingency (offering preferred food items) without addressing the critical consequence maintaining the aggression, which was distance from the aversive stimulus (the caregiver). The tiger displayed conflicted behavior, wanting the food but clearly wanting the person to leave, creating two competing contingencies.

The team shifted to a constructional approach using negative reinforcement. They created a controlled environment where the tiger could emit calm emotional behaviors (such as lying down while another caregiver provided food), while the person would briefly step forward and immediately step back before the tiger displayed aggressive responses. By providing the critical consequence (distance) contingent on various calm emotional behaviors (degrees of freedom), they directly addressed the underlying contingency rather than attempting to superimpose a new one.

Viewed through the framework of the five critical elements of the constructional approach described earlier in this paper, this case illustrates each component in practice. The team identified the terminal repertoire, a calm, affiliative interaction between the tiger and the previously aversive caregiver, and analyzed the current relevant repertoire, noting that aggressive displays were maintained by increased distance. The change procedures focused on reinforcing calm emotional behaviors with that same critical reinforcer (distance), thereby preserving effective behaviors while expanding the tiger’s degrees of freedom to include multiple, less costly alternatives. As these contingencies were addressed, the program transitioned to positive reinforcement for ongoing husbandry and medical care, ensuring that maintaining consequences continued to support welfare and engagement. The final element, progress monitoring, was embedded throughout the process, as sessions were documented and analyzed to guide each training refinement.

The results were efficient and effective. After eight sessions totaling 42 min of training, the tiger’s behavior toward the caregiver measurably improved. Instead of displaying aggression, the tiger began to “chuff” (an affiliative tiger vocalization) when hearing this caregiver approach and showed anticipation rather than fear or aggressive responses. The transformation was comprehensive, allowing the caregiver to conduct medical training sessions, including giving injections, and even lead educational presentations for guests, all with an animal that previously could not tolerate his presence.

This case study powerfully demonstrates several key principles of the constructional approach: (1) the importance of identifying and working with the critical consequence maintaining behavior rather than against it, (2) the efficiency of addressing the underlying contingency rather than merely superimposing a new one, and (3) the potential for rapid, measurable improvements in previously intractable situations when applying this framework correctly.

**Muskox (*Ovibos moschatus*): Transforming Aggressive Responses Towards Caregivers**

As described by Pedersen [33], a female muskox at Copenhagen Zoo, in Copenhagen, Denmark, had displayed long-standing aggressive behavior towards caregivers, charging the fence and hitting it with her horns whenever staff approached. Traditional attempts to counter-condition this behavior by offering highly valued food (carrots) had failed, as the muskox continued to display aggressive responses despite the presence of preferred food items.

NCA revealed that the muskox’s behavior was maintained by negative reinforcement; caregivers typically moved away after aggressive displays, reinforcing the charging behavior. The traditional approach had attempted to superimpose a positive reinforcement contingency without addressing the underlying negative reinforcement contingency.

The intervention used a similar constructional approach applied to the tiger case. The caregiver approached to a distance where the muskox emitted calm emotional behaviors, then immediately retreated before any aggressive responses were emitted. This process was repeated, gradually decreasing distance, and adding duration while reinforcing calm emotional behaviors with the critical consequence (distance) that maintained the aggressive response.

During the session, a behavioral shift occurred; the muskox began approaching the fence with her head up in what appeared to be curiosity rather than with her head down in an aggressive posture. This allowed the caregiver to toss carrots into the enclosure, which the muskox ate without displaying aggressive responses.

In a single session, the muskox’s behavior transformed. Routine, follow-up observations showed the muskox calmly taking food directly from the caregiver’s hands, reversing the previously entrenched aggressive pattern. The intervention achieved rapid, sustainable change with genuine assent by addressing the functional reinforcer maintaining the behavior rather than attempting to superimpose an unrelated contingency.

Beyond addressing fear and aggression, the constructional approach can also inform interventions for other behavior patterns that impact welfare, such as repetitive or stereotypic behaviors. For example, at the Santa Barbara Zoo in Santa Barbara, California, United States, a male emu (*Dromaius novaehollandiae*) was observed repeatedly pacing along a fence line during the breeding season [50]. Functional assessment revealed that the behavior was maintained by access to people, primarily staff members, and the opportunity to engage in courtship displays. Staff responded by introducing a stuffed burlap bag as a surrogate, allowing the emu to engage in courtship and mating behaviors. By providing access to this surrogate that fulfilled the same functional outcome before pacing began, staff addressed the pacing by rendering it unnecessary and increased engagement in a variety of calm, species-typical activities. The authors have also implemented constructional frameworks to address other examples of repetitive or stereotypic behavior in zoological settings; however, these applications have not yet been formally documented for inclusion in this manuscript.

#### 5.3.3. Common Principles Across Applications

Across the diverse case studies presented throughout this paper, several common principles of the constructional approach emerge that transcend specific behavioral contexts or species:

1. **Utilizing NCA:** Applying a systems-oriented framework to identify the full range of contingencies, maintaining behavior, including overlapping, competing, and superimposed arrangements, ensures interventions are grounded in the actual functional relations at work. This comprehensive analysis allows practitioners to see beyond superficial behavior patterns and understand the full matrix of contingencies that maintain them.
2. **Working With Functional Reinforcers:** Designing interventions that directly contact and use the reinforcers maintaining the animal’s current behavior, rather than superimposing unrelated contingencies. This principle acknowledges that functional reinforcers reveal valuable information about what matters to the animal and uses this information constructively rather than working against it.
3. **Initiating Interventions Under Appropriate Stimulus Conditions:** Effective interventions begin under stimulus conditions where the animal can emit desired behavioral responses. This principle acknowledges that accessing desired outcomes requires starting with conditions that ideally do not occasion unwanted responses. By carefully establishing an initial baseline in which desired responses are observed, constructional practitioners can systematically shape new repertoires through gradual approximations, while ensuring the animal retains the option to use the behavior that has historically produced successful outcomes, even if that behavior is not the caregiver’s preferred response.
4. **Providing Degrees of Freedom:** Creating multiple ways for animals to access important outcomes enhances welfare and intervention effectiveness. Degrees of freedom serve multiple functions:
  - They provide a measure of coercion, revealing when animals have genuine choice versus when they are effectively forced into particular response patterns
  - They generate critical information about the animal’s preferences, abilities, and emotional state
  - They indicate when behaviors might be too difficult or aversive for the animal
  - They mitigate frustration, aggression, and fear by ensuring alternative pathways to reinforcement
  - They allow animals to effectively communicate “no” or “I’m uncertain” without missing reinforcement opportunities
  - They can reveal additional reinforcers operating in the environment, such as social reinforcement or the inherent reinforcing properties of learning and participation
5. **Building Repertoires with Genuine Assent:** Rather than using restraint, punishment, or other coercive techniques that may temporarily suppress behavior but cause additional distress, constructional approaches build behavioral repertoires that allow animals to participate in their care with genuine assent. This principle recognizes that behavior change achieved through compulsion or coercion often produces emotional side effects and is less durable than change built on a foundation of choice and functional contingencies. The speed with which animals often transform their behavior patterns under constructional programs demonstrates the effectiveness of this compassionate approach to behavior change.

These constructional principles are most effective when grounded in a nonlinear analysis of the contingencies maintaining the current behavior. Rather than focusing on eliminating surface-level behaviors, this approach enables practitioners to build durable new repertoires that give animals genuine options to access the outcomes they value. Working with, rather than against, the functional reinforcers maintaining an animal’s behavior produces measurable improvements in previously challenging situations across a wide range of species and contexts.

#### 5.3.4. Compassionate Framework Across Applications

The case studies presented throughout this section collectively illustrate how the constructional approach embodies compassion, as described by Scallan and Rosales-Ruiz [8], through both its methodology and outcomes. Across diverse species and contexts, three consistent elements of a compassionate framework emerge:

1. **Identifying Challenging Behaviors Without Judgment:** Rather than labeling animals’ responses as “problematic”, “aggressive”, or “non-compliant”, constructional interventions recognize these behaviors as competent strategies for navigating difficult conditions. Whether a gorilla avoiding a closing door or a muskox charging a fence, each behavior is understood as the product of specific contingencies rather than a flaw within the individual. This nonjudgmental perspective creates conditions for effective intervention without blame or coercion.
2. **Building Empathy by Analyzing Contingencies from the Animal’s Perspective:** Using NCA, constructional practitioners identify the critical outcomes that are meaningful to the animal, such as distance from aversive stimuli, access to social companions, or opportunities for preferred sensory experiences such as touch, sound, or visual engagement. This shift from asking “How do we stop this behavior?” to “What desirable outcomes are maintaining this behavior?” was evident in cases such as the sheep who sought goat companionship and the rhinoceros for whom multiple reinforcement pathways were constructed. Through this process, interventions are organized around the animal’s needs and perspectives, rather than human convenience.
3. **Taking Action to Build Behavioral Repertoires That Enhance Well-Being:** The interventions consistently created conditions in which animals could build new repertoires that provided multiple paths to access critical reinforcers. From the parrot flock that transitioned from fearful avoidance to eagerly approaching, to the tiger that learned to engage comfortably with a previously aversive caregiver, these case studies illustrate how constructional programs create genuine choice through increased degrees of freedom. By expanding, rather than suppressing, behavioral options, the constructional approach enhances animal well-being while building steady progress towards training goals.

Together, these elements reflect the “shifts in functional relationships… from aversive to increasingly appetitive contexts” described by Kaplan-Reimer et al. [7]. The effectiveness and efficiency of these interventions, often achieving in minutes what traditional methods failed to accomplish in months, further emphasize how a constructional, compassionate framework benefits both animals and caregivers. This approach supports institutional goals for optimal welfare by fostering meaningful, sustainable human–animal relationships grounded in functional understanding and intentional development of well-being, hallmarks of a compassionate practice. 

## 6. Implementation in Zoo Settings

### 6.1. Practical Entry Points for Individual Practitioners

This section translates constructional principles into practical guidance for zoo professionals, offering strategies for both individual trainers and institutions.

Zoo professionals interested in implementing a constructional approach can begin with several practical and immediately applicable strategies:

1. **Focus on Building Repertoires:** Define training goals in terms of what desirable behaviors the animal should learn to emit, rather than focusing on stopping undesired behaviors. Ask, “What does this animal need to learn to do instead?”
2. **Use Reinforcement Based on Functional Contingency Assessment:** Deliver reinforcement based on an understanding of the critical consequences maintaining behavior, not on procedural hierarchies or personal preference. Recognize when an animal’s responding indicates that negative reinforcement (access to distance) or positive reinforcement (access to appetitive stimuli) is maintaining behavior, and design interventions accordingly.
3. **Start Under Conditions That Support Desired Responses:** Arrange stimulus conditions so that animals are likely to emit desired behaviors from the beginning of the training process. Prevent conditions that might lead practitioners to rely on the use of extinction, DRI/DRA, exposure therapies, or punishment procedures and avoid their use altogether, as they can produce emotional distress and unsustainable outcomes.
4. **Incorporate Genuine Choice, Honor Ongoing Assent, and Increase Degrees of Freedom:** Design training contexts so that animals have multiple ways to access critical reinforcers, thereby providing genuine choice [15]. To honor assent, ensure that animals have more than one functional behavioral path to critical outcomes, not only at the start of an interaction but maintained throughout the training process. Ongoing availability of multiple access options is essential for assent to remain valid and for interventions to align with constructional principles [42,44].
5. **Address Emotional Welfare by Modifying Contingencies:** Rather than attempting to directly counter-condition or extinguish emotional responses, change the contingencies that occasion the behavior. For example, if fear or aggressive behaviors are observed, arrange conditions (such as distance) to reinforce calm emotional responding [28,49].

### 6.2. Strategies for Organization-Level Implementation

For organizations within the zoo and aquarium community aiming to adopt a constructional approach more broadly, the following strategies are recommended:

1. **Comprehensive Staff Training:** Provide training in constructional principles to all animal care staff, focusing on the application of NCA, repertoire building, and degrees of freedom, rather than merely procedural teaching.
2. **Protocol Review Through a Constructional Lens:** Evaluate and revise existing training and husbandry protocols to shift from reduction-based strategies to constructional, repertoire-building practices.
3. **Behavior-Based Welfare Monitoring:** Develop welfare assessment tools that measure observable indicators of emotional behaviors, degrees of freedom, and repertoire expansion rather than relying solely on the absence of problem behaviors.
4. **Collaborative Learning and Professional Development:** Encourage participation in constructional training communities, academic resources, learning platforms, and other professional forums that support continued education and dialog on the constructional approach.
5. **Case Study Documentation and Sharing:** Document constructional interventions and share case studies within and beyond the organization. Systematic documentation not only strengthens internal learning but also contributes to the advancement of best practices across the zoo and aquarium community.

## 7. Benefits of the Constructional Approach

Implementing a constructional approach in zoological settings offers numerous interconnected benefits:

- **Enhanced Welfare Outcomes:** By focusing on building desirable behavioral repertoires rather than eliminating undesired behaviors, animals experience greater agency, reduced stress, and improved emotional well-being.
- **Durable, Sustainable Behavior Change:** Addressing functional reinforcers, rather than layering superimposed contingencies, produces behavioral change that is more rapid, durable, and resistant to relapse [33,37].
- **Expanded Behavioral Skills:** Animals develop broader and more flexible repertoires, increasing their ability to navigate complex and dynamic environments.
- **Compassionate, Welfare-Centered Management:** The constructional approach avoids reductive procedures, promotes genuine choice, and aligns with public expectations for ethical and evidence-based animal care practices.
- **Individualized Interventions:** Rather than relying on generalized recipes, practitioners apply NCA to create customized interventions tailored to the needs of each animal.
- **Reduction in Emotional Fallout:** By increasing degrees of freedom, the approach minimizes the emotional side effects often associated with extinction, punishment, and coercive methods.
- **Increased Training Efficiency:** Constructional methods often achieve meaningful behavior change in measurably short timeframes compared to traditional methods, as demonstrated across numerous case examples [15,33].
- **Improved Staff Safety:** Addressing aggressive and fear-based behavior at the functional contingencies level improves staff safety without relying on compulsion or suppression.
- **Strengthened Human–Animal Relationships:** Prioritizing understanding of functional contingencies fosters more profound empathy and creates relationships based on genuine assent and enhanced communication.

### Future Learning and Continued Growth

This article introduces the constructional approach as a framework for improving animal well-being in zoological settings. The examples included are intended to illustrate key principles and occasion reflection rather than serve as prescriptive protocols. While many of the practical examples come from the authors’ documented case studies and professional presentations, they represent a subset of a broader and growing body of applications. Numerous additional examples across a wider range of taxa and managed care contexts have been implemented in practice but remain undocumented in formal literature at this time.

Due to the limited availability of published case studies applying the constructional approach in zoological contexts, the authors have drawn from their own work and experiences to highlight foundational applications and outcomes. These examples are not exhaustive but rather intended to provide a springboard for further exploration. It is anticipated that as interest in constructional principles expands and fluency in their application increases, more documented examples from a wider range of practitioners and species will become available. The authors actively support and encourage the dissemination of such work through presentations, peer-reviewed publications, and professional dialog.

Although the case studies presented here reflect successful outcomes, the narrative format and qualitative nature of this review limit causal conclusions. The authors acknowledge that the examples represent documented successful applications and may not capture unsuccessful or incomplete implementations. Future research should therefore prioritize systematic evaluation, standardized welfare measures, and investigation of potential barriers to implementation, including access to specialized training, philosophical alignment with assent-based practices, and the challenges of operationalizing and objectively measuring the principles and their applications. Recognizing these limitations underscores the need for continued empirical inquiry to complement and strengthen the growing foundation of evidence.

Readers are invited to engage with the expanding constructional community, share their own case examples, and contribute to a collective understanding of how these principles can support sustainable behavior change and optimal animal well-being across diverse settings. Through continued study, practice, and shared inquiry, the application of constructional strategies will continue to evolve and strengthen throughout the field.

## 8. Conclusions

The constructional approach offers a scientifically grounded, compassionate framework for enhancing zoo animal welfare management. By focusing on building behavioral repertoires rather than eliminating problems, utilizing sophisticated contingency analysis, incorporating genuine choice, and addressing emotional welfare through contingency management, zoos can develop more effective and welfare-centered animal care practices.

A key strength of the constructional approach is its ability to identify and address functional reinforcers through NCA, which helps practitioners recognize when interventions might merely superimpose a new contingency rather than addressing the underlying one. As Pedersen [33] demonstrated with examples involving tigers, muskox, and parrots in zoo settings, addressing the functional reinforcer (often distance for fear or aggression) rather than superimposing a different contingency leads to more rapid, durable, and welfare-enhancing outcomes.

As the zoo and aquarium communities continue to evolve their focus toward optimal animal welfare as both a means and an end, the constructional approach provides a valuable set of principles and practices that align with this mission. It represents a demonstrable advancement over previous behavior intervention models, including hierarchical approaches that prescribe sequential application of techniques without adequate consideration of behavioral function [16,17,18,19,20]. While hierarchical models often lead to delays in effective intervention, coercive applications of techniques, and failure to address the functional relationships maintaining behavior, the constructional approach offers individualized, function-based interventions that optimally maximize benefits and minimize harms to animals.

By learning from the growing body of evidence and expertise in this area, zoo professionals can enhance their capacity to care for animals in ways that promote both welfare and conservation outcomes. The constructional approach represents not a passing trend but a substantive and enduring contribution to animal welfare science, one that has particular relevance for the complex challenges of managing diverse species in zoological settings. Though illustrated here through zoo-based examples, the framework is broadly applicable across aquariums, sanctuaries, rehabilitation centers, and research facilities. By adopting a constructional approach, organizations in the zoo community can lead with compassion, creating environments where animals experience meaningful choice, reduced coercion, and lasting improvements in well-being.

## Figures and Tables

**Table 1:** Six common emotions and their related contingencies.

Emotion	Contingency Description
Fear	A distancing contingency where moving away from an aversive stimulus is maintained by negative reinforcement.
Anger/Aggression	A distancing contingency where driving an aversive stimulus away is maintained by negative reinforcement.
Panic	A nonspecific distancing contingency where the animal moves away from an aversive stimulus, maintained by negative reinforcement.
Anticipation/Interest	A nearing contingency maintained by positive reinforcement.
Conflict/Hesitation	Competing contingencies where both positive and negative reinforcement are operating simultaneously.
Anxiety/Stress	Competing contingencies where the animal lacks the behavioral repertoire or skills to effectively access positive reinforcement, resulting in a negative reinforcement contingency.

## Data Availability

No new data were created or analyzed in this study. Data sharing is not applicable to this article.

## Abbreviations

The following abbreviations are used in the manuscript:

NCA	Nonlinear Contingency Analysis
CET	Constructional Exposure Therapy
DRA	Differential Reinforcement of Alternative Behavior
DRI	Differential Reinforcement of Incompatible Behavior
ABC	Antecedent Behavior Consequence
EAZA	European Association of Zoos and Aquaria
